# The inhibition of IRE1alpha/XBP1 axis prevents EBV-driven lymphomagenesis in NSG mice

**DOI:** 10.1128/spectrum.02636-23

**Published:** 2023-10-26

**Authors:** Andrea Arena, Maria Anele Romeo, Agnese Po, Rossella Benedetti, Maria Saveria Gilardini Montani, Roberta Gonnella, Roberta Santarelli, Aurelia Gaeta, Enrico De Smaele, Mara Cirone

**Affiliations:** 1 Department of Experimental Medicine, Sapienza University of Rome, Rome, Italy; 2 Department of Molecular Medicine, Sapienza University of Rome, Rome, Italy; Barnard College, Columbia University, New York, New York, USA

**Keywords:** EBV, lymphomagenesis, IRE1alpha/XBP1s, NSG mice

## Abstract

Post-transplant lymphoproliferative disorders (PTLD) are a group of heterogeneous diseases originating in conditions of immune deficiency, whose main driver is considered to be Epstein-Barr virus (EBV). Here, we explored the role of IRE1alpha/XBP1s axis in EBV-driven lymphomagenesis in an NOD SCID gamma mouse model, as these animals develop malignancies that closely resemble PTLD when engrafted with EBV-positive peripheral blood momonuclear cells (PBMC). This study evidences for the first time that 4μ8C IRE1 alpha endoribonuclease inhibitor prevented lymphomagenesis *in vivo* and B-cell immortalization *in vitro* driven by the virus. At the molecular level, 4μ8C reduced the expression of EBV antigens such as ZEBRA and LMP1, downregulated c-Myc and cyclin D1, and prevented the activation of STAT3, molecules known to be involved in viral lymphomagenesis. The results obtained in this study suggest that the inhibition of IRE1 alpha endoribonuclease may represent a promising therapeutic strategy to prevent EBV-associated PTLD that arise in immune-deficient patients.

**IMPORTANCE:**

The novelty of this study lies in the fact that it shows that IRE1 alpha endoribonuclease inhibition by 4μ8C was able to counteract Epstein-Barr virus-driven lymphomagenesis in NOD SCID gamma mice and prevent B-cell immortalization *in vitro,* unveiling that this drug may be a promising therapeutic approach to reduce the risk of post-transplant lymphoproliferative disorders (PTLD) onset in immune-deficient patients. This hypothesis is further supported by the fact that 4μ8C impaired the survival of PTLD-like cells derived from mice, meaning that it could be helpful also in the case in which there is the possibility that these malignancies have begun to arise.

## OBSERVATION

**Fig 1:**
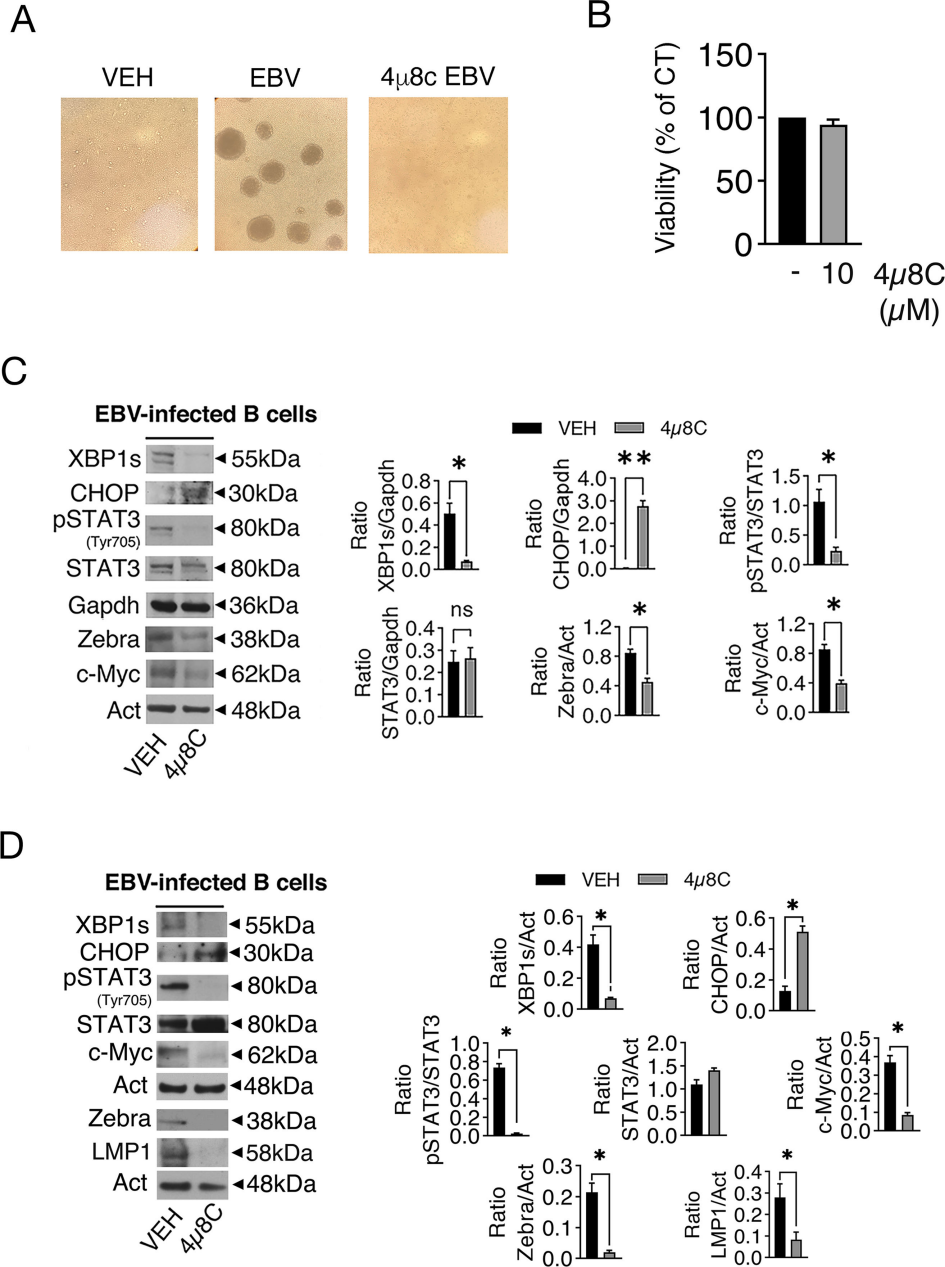
IRE1alpha/XBP1s axis is required for EBV-induced B-cell *in vitro* immortalization. (**A**) Optical microscope images showing LCL formation induced by EBV in the absence and in the presence of 4µ8C. (**B**) Cell viability of primary B lymphocytes infected by EBV and treated or not by 4µ8C (10 µM). (**C**) XBP1s, ZEBRA, pSTAT3 (Tyr705), STAT3, CHOP, c-Myc, and LMP1 expression, as evaluated by western blot analysis in primary B lymphocytes following 96 hours and (**D**) 15 days of EBV-infection; actin (Act) or GAPDH were used as loading controls and one representative experiment is shown. The histograms represent the densitometric analysis of the ratio of specific protein/actin or GAPDH. Data are represented as the mean + SD of three independent experiments or technical replicates. *P < 0.05.

**Fig 2:**
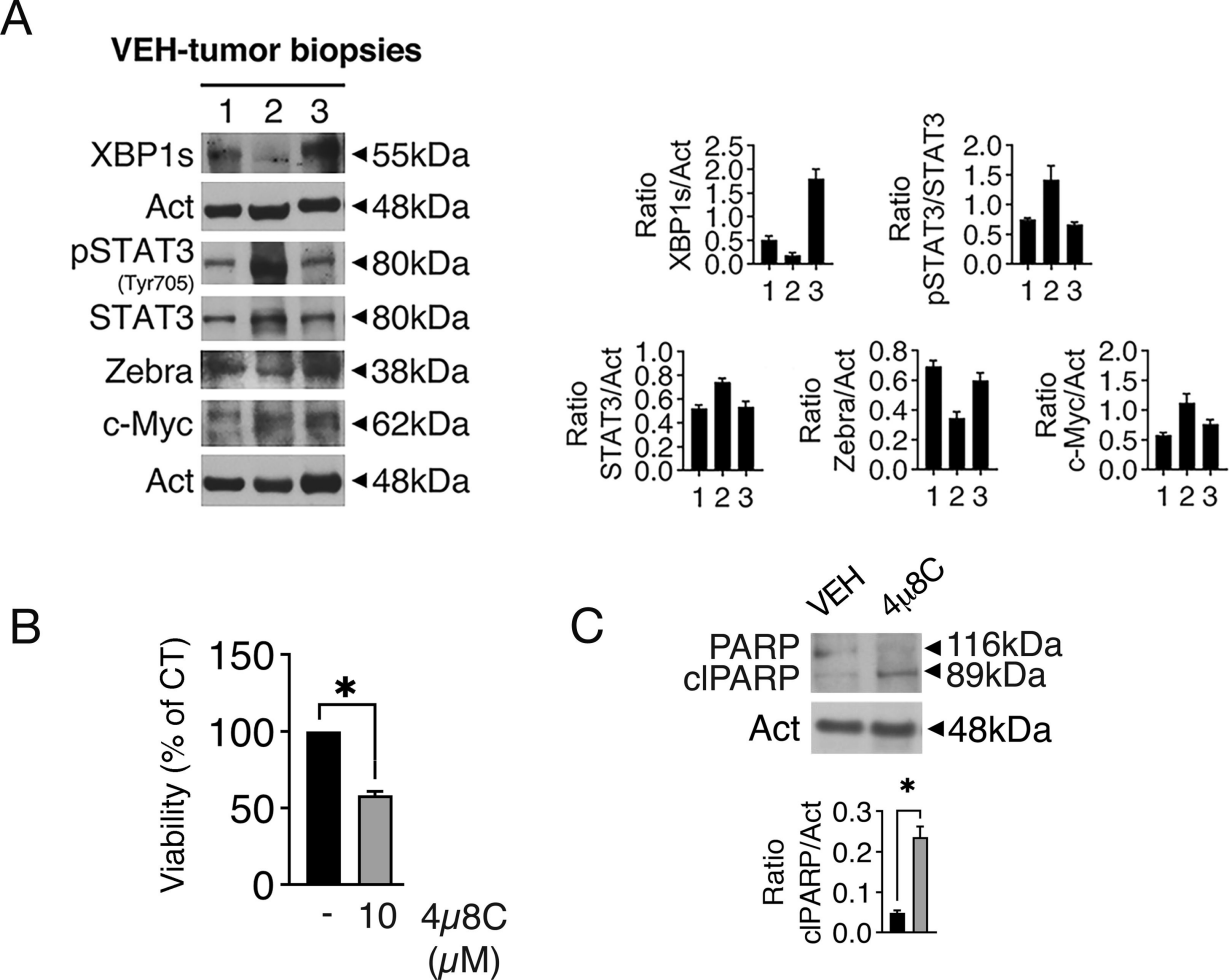
Tumor cells derived from NSG mice express XBP1s, ZEBRA, pSTAT3 (Tyr705), and c-Myc and are susceptible to 4µ8C-induced cytotoxic effect. (**A**) Expression of XBP1s, ZEBRA, pSTAT3 (Tyr705), and c-Myc by fresh tumor biopsies derived from three different vehicle-treated mice after 34 (lane 1), 52 (lane 2), or 74 (lane 3) days of treatment. Actin (Act) was used as a loading control and one representative experiment is shown. The histograms represent the densitometric analysis of the ratio of specific protein/actin. Data are represented as the mean + SD of three independent experiments or technical replicates. (**B**) Cell viability of PTLD-like cells derived from NSG-mice treated or not by 4µ8C (10 µM) and counted by trypan blue exclusion assay. The histograms represent the mean percentage of cell viability relative to the control plus SD of three independent experiments. *P < 0.05. (**C**) Expression level of clPARP as evaluated by western blot analysis in PTLD-like cells derived from NSG mice and treated or not by 4µ8C. Actin (Act) was used as a loading control and one representative experiment is shown. The histograms represent the mean of the densitometric analysis of the ratio between the protein and the appropriate control. Data are represented as the mean + SD of three independent experiments. *P < 0.05.

Post-transplant lymphoproliferative disorders (PTLD) are heterogeneous diseases mainly deriving from the transformation of germinal center B cells and their descendants, including plasma cells (1, 2). Epstein-Barr virus (EBV), a gammaherpesvirus strongly involved in human cancer, is considered the main driver of PTLD, being detected in the majority of these malignancies, with viral replication occurring in 60% of cases (3). Besides latent proteins such as LMP1 and EBNA2, viral lytic antigens are involved in EBV-driven lymphomagenesis. Indeed, it has been reported that lymphoblastoid cell lines (LCLs) immortalized by EBV mutants *BZLF1*-deleted (Z-KO) or *BRLF1*-deleted (R-KO) are less efficient in promoting PTLD in severe combined immunodeficiency (SCID) mice (4). PTLD express the spliced form of X-box-binding protein 1 (XBP1s), originating from the endoribonuclease activity of inositol-requiring enzyme 1alpha (IRE1alpha) on XBP1 (1). This is the most evolutionarily conserved arm of unfolded protein response (UPR), which also includes protein kinase RNA-like endoplasmic reticulum kinase and activating transcription factor (ATF) 6. XBP1s is required in plasma cell differentiation (5), a process that has been shown to trigger EBV replication *in vivo* (6). Moreover, XBP1s can directly activate lytic gene expression in EBV-transformed LCLs (7). Notably, EBV lytic antigens, plasma cell differentiation, and XBP1s expression can be concomitantly detected in PTLD and identify a high-risk subgroup of patients (8). In addition to these molecules, PTLD cells often display the deregulated expression of c-Myc (9), an oncogene that cooperates with XBP1s in promoting carcinogenesis (10). Notably, IRE1alpha/XBP1s axis is also interconnected with STAT3 (11), a pathway strongly involved in lymphomagenesis as well as in EBV-mediated B-cell *in vitro* immortalization (12, 13). Given the important role of XBP1s in cancer and EBV lifecycle, here we investigated whether the inhibition of IRE1alpha/XBP1s axis by 4μ8C (Fig. S1A) could prevent the outgrowth of EBV-driven malignancies in NOD SCID gamma (NSG) mice (14). At this aim, mice were reconstituted with PBMCs derived from EBV-positive donors and treated with 4μ8C (50 mg/kg of body weight) or vehicle (VEH) from day 7 to sacrifice (every other day). The animals were sacrificed when displaying suffering signs or, at most, after 95 days of treatment. Autopsies revealed that mice treated with 4μ8C did not develop tumors, while solid tumors were detected in all except one vehicle-treated mice (Table 1; Fig. S1B). We found that PTLD-like cells derived from NSG mice, freshly isolated from tumor tissues by mechanic disaggregation, expressed CD19 B-cell marker (Fig. S1C), harbored EBV DNA (Fig. S1D), and expressed several viral antigens such as EBNA1, EBNA2, LMP1, LMP2a, BZLF1 (ZEBRA), and, in some cases, GP220 (Table 1). Next, we evaluated whether 4μ8C could prevent B lymphocyte *in vitro* immortalization driven by EBV. As shown in Fig. 1A, we found that this drug was also able to impair LCL formation, although it was not cytotoxic against primary B lymphocytes (Fig. 1B), suggesting that it was preventing viral-mediated immortalization. At the molecular level, we observed that 4μ8C reduced the expression of XBP1s, p-STAT3 (Tyr 705), c-Myc as well as ZEBRA in B lymphocytes 96 hours post *in vitro* EBV infection (Fig. 1C), while it upregulated the pro-apoptotic UPR molecule CHOP (Fig. 1C). The latter has been reported to cause death of premalignant cells in a mouse model of lung cancer driven by *G12V* mutant Kras and, therefore, its increased expression in 4μ8C-treated B cells could contribute to carcinogenesis prevention (15). The molecular changes mediated by 4μ8C, still observed 15 days post-infection of B lymphocytes, together with the downregulation of the latent antigen LMP1 (Fig. 1D), could play a key role in counteracting EBV-driven lymphomagenesis. This hypothesis may also be supported by the finding that fresh tumor biopsies expressed XBP1s, ZEBRA, p-STAT3 (Tyr 705), and c-Myc (Fig. 2A). Although in previous studies it has been shown that the inhibition of IRE1alpha RNase activity could reduce the growth of c-Myc-overexpressing lymphoma cells (16), including those associated with EBV (17), given that UPR activation can help cells to face the stress caused by oncogene hyper-activation (18), this is the first study showing that 4μ8C may prevent lymphomagenesis *in vivo* as well as B-cell immortalization *in vitro* driven by EBV. Moreover, as we found that this drug reduced the survival of PTLD-like cells, freshly isolated from tumors arising in mice after 34, 52, and 74 days of VEH treatment (Fig. 2B) and caused apoptotic cell death in these cells (Fig. 2C), it may be hypothesized that 4μ8C could be useful also in the cases in which EBV viral load starts to rise and there is a suspicion of PTLD onset.

**TABLE 1:** Time and site of tumor localization and EBV gene expression of tumors arising in NSG mice^*a*^

Treatment	No.	Day	Solid tumor	EBV gene expression
VEH	1	74	Abdomen	EBNA1, EBNA2, LMP2A, LMP1, BZLF1, GP220
	2	52	Abdomen, liver	EBNA1, EBNA2, LMP2A, LMP1, BZLF1
	3	52	Liver, spleen	EBNA1, EBNA2, LMP2A, LMP1, BZLF1, GP220
	4	34	Abdomen, liver	EBNA1, EBNA2, LMP2A, LMP1, BZLF1
	5	95	None	N/A
4µ8C	1	95	None	N/A
	2	95	None	N/A
	3	95	None	N/A
	4	76	None	N/A
	5	95	None	N/A
Negative control (HCoEpC)	–	–	–	N/D
Positive control (B95-8)	–	–	–	EBNA1, EBNA2, LMP2A, LMP1, BZLF1, GP220

^*a*^ N/A, not applicable; N/D, not detected. See Observation for details.

## Data Availability

All data that support the findings of this study are available from the corresponding author (mara.cirone@uniroma1.it) upon reasonable request.

## SUPPLEMENTAL MATERIAL

Supplemental material spectrum.02636-23-s0001.docx Supplemental methods and Fig. S1.

ASM does not own the copyrights to Supplemental Material that may be linked to, or accessed through, an article. The authors have granted ASM a non-exclusive, world-wide license to publish the Supplemental Material files. Please contact the corresponding author directly for reuse.
