# Supplementary material for: The inhibition of IRE1alpha/XBP1 axis prevents EBV-driven lymphomagenesis in NSG mice
Source: Microbiol Spectr. 2023 Oct 26;11(6):e02636-23. doi: 10.1128/spectrum.02636-23 (PMC10715178; doi:10.1128/spectrum.02636-23)
Supplement: Supplemental material — Supplemental methods and Fig. S1. [file spectrum.02636-23-s0001.docx]

**Materials and methods**

**Animals**

NSG male mice (614NSG) (Charles River, Massachusetts, USA) were housed in individually ventilated clear plexiglas cages (20 × 22 × 20 cm, Tecniplast, Buguggiate (VA) Italy) under standard laboratory conditions at a temperature of 22 ± 2°C, 70% humidity, 12 hours (h) light/dark cycle and free access to food and water. All efforts were made to minimize the number of animals used in the study and their suffering and the experiments were performed in strict compliance with animal welfare National Laws (D.lgs. 26/2014), European Communities Council Directives (n. 2010/63/UE) and with the formal approval of the local [“Organismo Preposto al Benessere degli animali” (O.P.B.A.), University of Rome Sapienza] and national (Ministry of Health) animal care committees. Animal experiments have been registered as legislation requires (Authorization from the Ministry of Health n° 613/2021-PR). A veterinary surgeon was present during the experiments. Animal care, before and after the experiments, was carried out only by trained personnel.

**Mice treatments**

Mice were injected intraperitoneally (i.p.) with 20 × 10^6^ EBV-positive PBMCs in PBS (Aurogene, Rome, Italy). From day 7 to sacrifice, groups of NSG mice (n=5) were treated by oral gavage with 4μ8C (8-formyl-7-hydroxy-4-methylcoumarin) (50 mg/kg) (1) (MedChem Express, Monmouth Junction, NJ, USA, HY-19707) or vehicle only (VEH) (DMSO (10%) +PEG 300 (40%) + [Tween-80](about:blank) (5%) + saline (45%)), administered every other day. 4μ8C is a potent and selective IRE1 Rnase inhibitor acting by blocking the substrate access of IRE1’s active site and selectively inactivates both XBP1 splicing and IRE1-mediated mRNA degradation.

**Cell extraction from mice tumor**

Mice were sacrificed by cervical dislocation, the tumor tissue was surgically removed and transferred in a cell culture dish containing PBS, mechanically disaggregated and stored at − 80°C until utilization.

**B lymphocyte isolation, treatment and EBV infection**

B lymphocyte derived from human peripheral blood mononuclear cells (PBMCs) isolated by Ficoll-Paque gradient centrifugation (Lympholyte; Cedarlane, CL5020) from buffy coats of healthy donors. B lymphocytes were isolated from PBMCs by immunomagnetic cell separation using anti-CD19-conjugated microbeads according to the manufacturer's instructions (Miltenyi Biotec, 130–050-301) and cultured in RPMI-1640 (Sigma-Aldrich, St Louis, MO, USA, R0883), 10% fetal bovine serum (FBS) (Sigma-Aldrich, F7524), L-glutamine (Aurogene, AU-X0550), streptomycin (100 µg/ml) and penicillin (100 U/ml) (Aurogene, AU-L0022), complete medium (CM), in 5% CO_2_-saturated humidity at 37°C. B cells were pre-treated or not with 4μ8C (10 μM) (Sigma-Aldrich, Saint Louis, MO, USA, SML0949) for 30 minutes, infected with EBV at a multiplicity of infection (MOI) of 10 genome equivalents/cells, for 30 min at 37°C and cultured for 96 h or 15 days in the absence or in the presence of 4μ8C (10 μM) added every two days. Each experiment was performed in triplicate and repeated at least three times. EBV was produced by B95.8 cells treated with phorbol 12-myristate 13-acetate (TPA) (30 ng/mL) and sodium butyrate (3mM) for 96 h and cell supernatant was concentrated 200X by ultracentrifugation at 29000 rpm for 1h and 30 minutes.

**Immunofluorescence and FACS analysis**

Cells isolated from tumor biopsies derived from vehicle-treated mice were stained with anti-human CD19-FITC primary antibody (Milteneyi Biotech, 130-113-168) and anti-human IgG-FITC (Milteneyi Biotech ,130-118-340) and analyzed by FACSCalibur, using CELLQuest software (BD Biosciences, San Josè, CA, USA) At least 10 × 10^3^ events were acquired for each sample.

**EBV-transformed LCLs culture and treatment**

For EBV-transformed LCLs, 50 × 10^6^ PBMCs were infected at multiplicity of infection (MOI) of 10 genome equivalent/cell for 2 hours at 37 °C, then seeded in 24-well plates at 1 × 10^6^/mL in 2 mL of complete RPMI 1640 supplemented with cyclosporine A (1 μg/mL) (Sigma Aldrich, St Louis, MO, USA; 30024) and treated with 4μ8C (10 μM) (Sigma-Aldrich, Saint Louis, MO, USA, SML0949) every two days. The research involving human subjects has been performed in accordance with the Declaration of Helsinki and has been approved by the ethic committee of Policlinico Umberto I, Rome, Italy (847/19).

**Quantitative real-time PCR**

Quantitative real-time PCR (qPCR) was performed in tumor tissue to detect and quantify the intracellular EBV DNA using ELITE MGB kit (ELITech) according to manufacturer’s instructions.

**RNA extraction and quantitative reverse transcription PCR (qRT-PCR)**

Total RNA was extracted from the frozen tumor tissues using TRIzol™ Reagent (Life Technologies, 15596026, Waltham, MA, USA) according to the manufacturer’s instructions. RNA was reverse transcribed using High-capacity cDNA Reverse Transcription Kit (Applied Biosystems, 4368814, Waltham, MA, USA) according to manufacturer’s instructions. Quantitative real-time PCR was performed, using the SensiFAST™ SYBR® No-ROX Kit (Bioline, London, UK). Primers used for the evaluation of gene expression are reported below:

| **Genes** | **Forwards (5’-3’)** | **Reverse (5’-3’)** |
| --- | --- | --- |
| *EBNA1* | TCGGCTTCTGGCGTGTGACC | CATAGCGTAAAAGGAGCAACA |
| *EBNA2* | TAACCACCCAGCGCCAATC | GTAGGCATGATGGCGGCAG |
| *LMP2A* | CTACTCTCCACGGGATGACTCAT | GGCGGTCACAACGGTACTAACT |
| *LMP1* | AATTTGCACGGACAGGCATT | AAGGCCAAAAGCTGCCAGAT |
| *BZLF1* | TCGCATTCCTCCAGCGATT | CAAGGACAACAGCTAGCAGACATT |
| *GP220* | CCTGTGTTATATTTTCACCACTTTC | ACCGCACCTGCAAGCA |
| *ACTIN* | TCATGAAGTGTGACGTGGACATC | CAGGAGGAGCAATGATCTTGATCT |

We used as a positive control the B95-8 cells, an EBV positive marmoset cell line, whereas as negative control the primary human colonic epithelial cells (HCoEpC). Each amplification was performed in triplicate and the average of three threshold cycles was used to calculate transcript abundance using the comparative quantification method based upon the ∆∆Ct method. The starting concentration of each specific product was divided by mean of the starting concentration of reference gene (*ACTIN*).

**Western blot analyses**

Samples were lysed in RIPA buffer and 15 µg of proteins were denatured and subjected to electrophoresis on 4–12% NuPAGE Bis-Tris gels (Life Technologies, Carlsbad, CA, USA) according to the manufacturer’s instruction. The gels were transferred to nitrocellulose membranes (Bio-Rad, Hercules, CA, USA) for 45 min in tris-glycine buffer. The membranes were washed and blocked in 1x PBS-0.1% Tween20 solution containing 2% of BSA (SERVA Electrophoresis GmbH, Heidelberg, Germany, 11946.02) for 1 h at RT, incubated with specific antibodies and developed using ECL Blotting Substrate (Advansta, San Jose, CA, USA).

**Antibodies**

To evaluate protein expression the following primary antibodies were used: rabbit polyclonal anti-XBP1 (1:500) (Proteintech, Rosemont, IL, USA, 24868-1-AP), mouse monoclonal anti-ZEBRA (1:100) (Santa Cruz Biotechnology Inc, Dallas, TX, USA, sc-53904), rabbit polyclonal anti-pSTAT3 Tyr705 (1:500) (Cell Signalling, Danvers, MA, USA, 9145), mouse monoclonal anti-STAT3 (1:500) (Santa Cruz Biotechnology Inc, Dallas, TX, USA, sc-482), rabbit polyclonal anti-CHOP (1:1000) (Proteintech, 15204-1-AP, Rosemont, IL, USA), rabbit polyclonal anti-c-MYC (1:500) (Proteintech, Rosemont, IL, USA, 10828-1-AP), mouse monoclonal anti-LMP1 (clone S12) (1:100) (Sigma-Aldrich, Saint Louis, MO, USA, MABF2248) and rabbit polyclonal anti-PARP (1:500) (Cell Signaling, Danvers, MA, USA, 9542). Mouse monoclonal anti-β-actin (1:10000) (Sigma-Aldrich, Burlington, MA, USA, A5316) and mouse monoclonal anti-GAPDH (1:10000) (Santa Cruz Biotechnology Inc, Dallas, TX, USA, sc-137 179) were used as loading control. The goat anti-Mouse IgG Peroxidase Conjugate (Sigma-Aldrich, Burlington, MA, USA, 401215) and the goat anti-Rabbit IgG Peroxidase Conjugate (Sigma-Aldrich, Burlington, MA, USA, DC03L) were used as secondary antibodies.

**Cell Assay Viability**

Primary B lymphocytes and PTLD-like cells derived from NSG-mice cells were treated for 24 h with 4μ8C (10 μM) (Sigma-Aldrich, Saint Louis, MO, USA, SML0949) and were counted by using light microscopy and trypan blue (Sigma-Aldrich, Burlington, MA, USA, 72571) dye exclusion assay. The experiment was repeated at least three times.

**Densitometric Analysis**

The quantification of protein bands was performed by densitometric analysis using the Image J software (1.47 version, NIH, Bethesda, MD, USA), which was downloaded from the NIH website (http://imagej.nih.gov (accessed on 10 February 2022).

**Statistical Analysis**

Data are expressed as the mean plus standard deviation (S.D.) of at least three independent experiments or technical replicates and statistical analyses were performed with Graphpad Prism® software (Graphpad software Inc., La Jolla, CA, USA). Student’s t test was used to demonstrate statistical significance between two groups. Difference was considered as statistically significant when *p*-value was: * < 0.05; ** < 0.01; *** < 0.001 and **** < 0.0001.


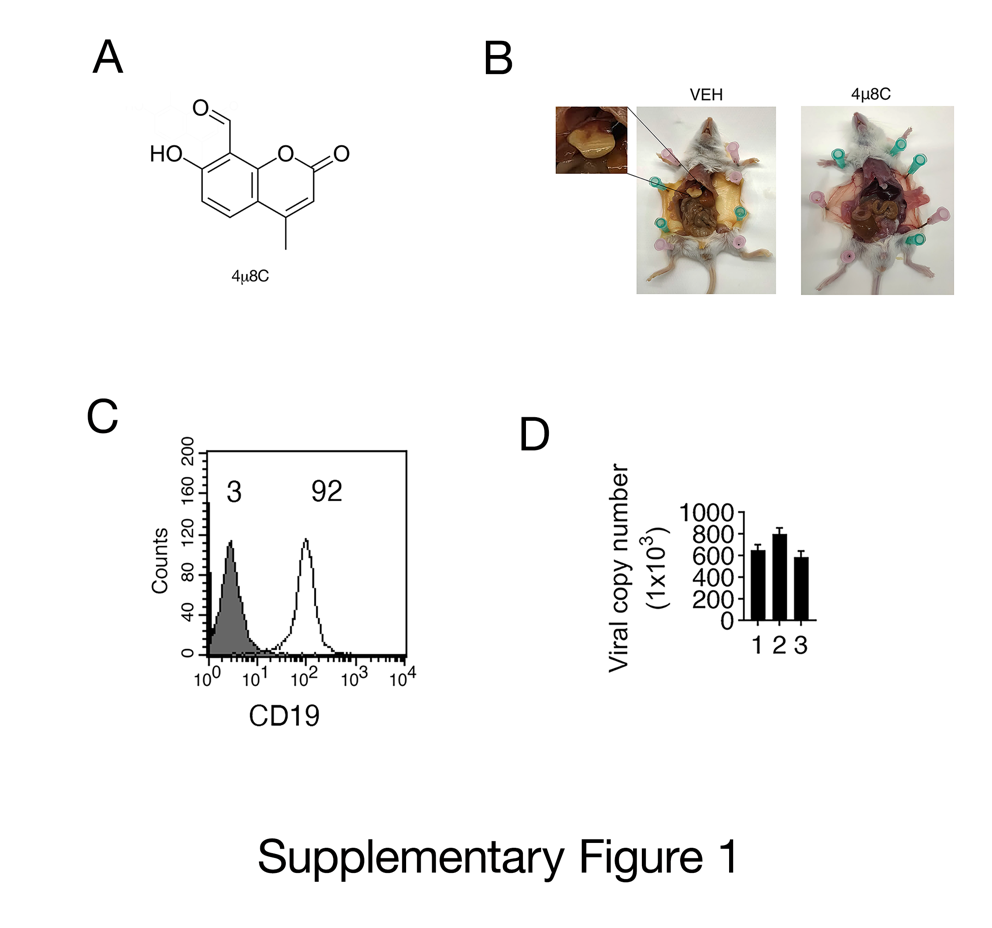


**Supplementary Figure 1. 4µ8C prevents EBV-driven PTLD-like formation in NSG mice**

(A) Chemical Structure of 4µ8C or 8-formyl-7-hydroxy-4-methylcoumarin; (B) representative macroscopic view of autopsies on VEH-treated or 4µ8C-treated mice; (C) FACS profile showing CD19 staining of PTLD-like cells originating from NSG mice; (D) EBV nuclear antigen 1 gene as amplified by qPCR in tumor biopsies derived from three different vehicle-treated mice. The histograms represent the mean plus S.D. of viral DNA copies of three technical replicates.

**References**

1. Nam ST, Park YH, Kim HW, Kim HS, Lee D, Lee MB, Kim YM, Choi WS. 2017. Suppression of IgE-mediated mast cell activation and mouse anaphylaxis via inhibition of Syk activation by 8-formyl-7-hydroxy-4-methylcoumarin, 4mu8C. Toxicol Appl Pharmacol 332:25-31.
